# Masking Ability of Monolithic and Layered Zirconia Crowns on Discolored Substrates

**DOI:** 10.3390/ma15062233

**Published:** 2022-03-17

**Authors:** Cristina Gasparik, Manuela Maria Manziuc, Alexandru Victor Burde, Javier Ruiz-López, Smaranda Buduru, Diana Dudea

**Affiliations:** 1 Department of Prosthetic Dentistry and Dental Materials, Iuliu Hatieganu University of Medicine and Pharmacy, 32 Clinicilor Street, 400006 Cluj-Napoca, Romania; gasparik.cristina@umfcluj.ro (C.G.); abv.alex@yahoo.com (A.V.B.); smarandabuduru@yahoo.com (S.B.); ddudea@umfcluj.ro (D.D.); 2 Department of Optics, Faculty of Science, Edificio Mecenas, Campus Fuente Nueva S/N, University of Granada, 18071 Granada, Spain; jruizlo@ugr.es

**Keywords:** zirconia, crown, color, masking ability

## Abstract

There is scarce information on the colorimetric behavior of monolithic and layered zirconia crowns in combination with various abutment colors. This study evaluated the masking ability on discolored substrates of monolithic and layered zirconia crowns. Anterior crowns were fabricated using 3Y-TZP zirconia and layering ceramic and divided into three groups: monolithic (ML), bi-layer (BL), and tri-layer (TL). The crowns were placed over eleven substrates (ND1-ND9, zirconia, metal), and CIE L*, a*, b*, C*, and h° color coordinates were measured in the cervical, middle, and incisal areas with a spectrophotometer. Masking ability was calculated using the color difference formula, and values were interpreted according to the perceptibility and acceptability thresholds. Data were analyzed statistically (α = 0.001). The L* coordinate was not significantly different between BL and TL crowns, regardless of the measurement area or substrate (*p* ≥ 0.001). In the middle area, the L* coordinate of the ML group was statistically different from the BL and TL groups only for zirconia and metal substrates, while in the incisal area, only for ND7 and metal substrates. The a* coordinate was significantly different between the ML and layered crowns for all measurement areas and substrates (except zirconia). The b* and C* coordinates differed significantly between the groups only in the cervical area (*p* < 0.001). The ML crown had better masking ability than the BL and TL crowns. However, the color differences for ML crowns were below the acceptability threshold for ND2, ND3, and ND7 substrates in the cervical and middle areas and below perceptibility threshold only for the incisal area. The lowest masking ability of the crowns was found for ND9 and metal substrates in all measurement areas.

## 1. Introduction

Tooth discoloration is a common clinical condition encountered in daily dental practice. It affects one or multiple teeth, and several factors may be involved in the etiology: caries and tertiary dentin formation, hemorrhage into the pulp chamber, endodontic procedures, and materials, as well as metabolic or idiopathic causes [1,2]. The esthetic improvement of tooth discoloration can be achieved by bleaching with oxidizing agents or prosthetic treatments when it is aimed to restoring the tooth with either a veneer or a full crown. Nevertheless, the procedure’s success is highly dependent on the skills and intuition of the dentist and dental technician since masking a discolored substrate is rarely a predictable process.

Today, there is an immense variety of dental materials available for the fabrication of indirect restorations. However, dental zirconia stands out because of its versatility, combining high strength with acceptable esthetics, allowing an entirely digitized fabrication process, and permitting additional individualization through conventional ceramic layering methods. 

The second generation of 3 mol% yttria-stabilized tetragonal zirconia polycrystal (3Y-TZP) zirconia has a reduced amount of aluminum oxide in its composition, compared to the first generation [3]. This material is sintered at higher temperatures, increasing grain size and reducing porosities, consequently improving the translucency. 3Y-TZP zirconia is indicated for monolithic or veneered restorations [4]. Traditional layering, over-pressing, file-splitting (CAD-on), and the cut-back technique are some veneering methods that can be combined with zirconia crowns [5].

Several factors influence the color appearance of zirconia restorations. Besides chemical composition and structure [3,6], other factors such as material thickness [5,7,8], processing parameters [9,10], shading technique and veneering material [11], substrate type and color [12,13,14,15,16], and luting agent [14,17,18,19] contribute to the overall color of the restoration.

According to the International Commission on Illumination (Commission Internationale de L’Éclairage—CIE), currently, the CIEDE2000 total color difference formula (ΔE_00_) [20], associated with the CIE L* a* b* color space, is widely implemented in clinical dentistry and dental research due to its better correlation with visual perception [21] and is recommended for total color difference computation by the International Standard Organization [22]. However, the use of the ΔE_00_ color difference formula alone is irrelevant unless the respective well-known visual 50:50% perceptibility and acceptability thresholds, determined in [23] and recommended in the latest guidance on color measurements for dentistry [22], are used for judging the significance of color differences [24].

In the past, the masking ability of a restorative material was evaluated using the color difference formulas (ΔE_ab_ or ΔE_00_), the translucency parameter (TP), or the contrast ratio (CR); notwithstanding, a recent systematic review concluded that the most appropriate method to assess the masking ability is using the color difference formula associated with the perceptibility (PT) and acceptability thresholds (AT) [25].

Several recent studies evaluated the masking ability and shade reproduction of dental materials [26,27,28,29,30]. The studies investigated the properties of monolithic samples when placed over discolored substrates. However, only a limited number and color of substrates were evaluated, while in clinical practice, the appearance of dental discoloration is highly variable.

Although extensive research has been recently conducted on the masking ability of restorative materials [12,13,14,15,16,17,18,19,31,32,33,34,35,36,37,38], there is still missing information on how color differences might impact visual perception when translucent restorations are evaluated over different discolored substrates. Furthermore, there is little information about the colorimetric behavior of monolithic and layered zirconia crowns in combination with various abutment colors. Most studies evaluated the masking ability using rectangular or disc samples [12,13,15,16,17,19,31,32,33,34,35,36,37,38], which do not reproduce the clinical conditions met in the oral cavity. 

Therefore, this study aimed to evaluate the masking ability of monolithic and layered zirconia crowns in each of the three areas (cervical, middle incisal) on eleven different discolored substrates. The null hypotheses were: (1) there were no significant differences in CIE L*, a*, b*, C*, and h° color coordinates between the monolithic and the layered zirconia crowns on the different discolored substrates; (2) the masking ability of monolithic and layered zirconia crowns on discolored substrates was acceptable.

## 2. Materials and Methods

This study used 3Y-TZP zirconia (Katana HT10, Kuraray Noritake Dental Inc., Tokyo, Japan) to fabricate anterior full coverage monolithic and layered crowns.

### 2.1. Crowns Fabrication

A phantom head’s upper right central incisor (DSE Expert, KaVo, Biberach, Germany) was prepared with a 1 mm circumferential chamfer finish line, 6° axial taper, 1 mm axial, and 1.5 mm incisal reductions. The prepared tooth was digitized using a laboratory scanner (InEos X5, Dentsply Sirona, Bensheim, Germany). 

**Figure 1:**
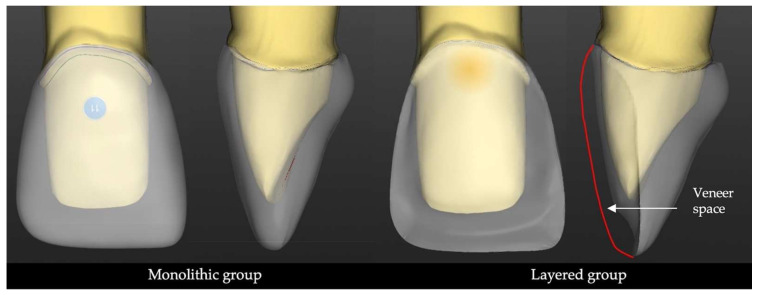
The 3D design of the monolithic and layered restorations in the CAD software.

Three-dimensional designs of the crowns were made with Exocad Dental CAD 2.4 (v.2.4, Exocad GmbH, Darmstadt, Germany) software. The following two designs were considered: a full-contour crown design with 1 mm labial thickness (monolithic group) and a partial veneer crown design (layered group) with 0.4 mm thickness of the framework on the labial surface and 0.6 mm space for the veneering ceramic (Figure 1). 

**Figure 2:**
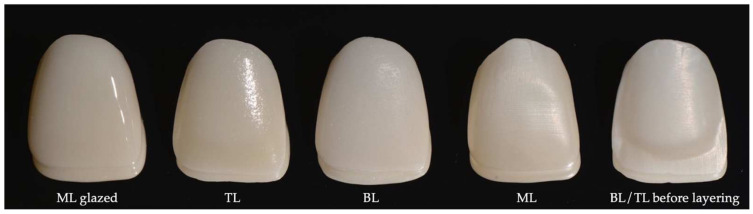
The appearance of the restorations in different fabrication steps.

The restorations were dry-milled under continuous vacuuming (Imes iCore 250i, Imes iCore GmbH, Eiterfeld, Germany) using a 3Y-TZP zirconia blank and then sintered at 1500 °C for 2 h (Mihm Vogt HT2, GmbH, Stutensee, Germany). The finishing of the sintered crowns was done using silicone discs (Meister SC51, Kuraray Noritake Dental Inc., Tokyo, Japan). The labial surface of the layered group was sandblasted using aluminum oxide (50 microns, 2 bars), and impurities were removed from the crown surfaces by immersing the restorations in an ultrasonic cleaner with distilled water for 10 min (Figure 2).

The layered group was further divided into two groups according to the ceramic layers applied: bi-layer group with 0.6 mm enamel layer (BL) and tri-layer group with 0.3 mm dentin and 0.3 mm enamel layers (TL). For the BL group, a 0.8 mm thick enamel veneering ceramic (CZR Cerabien Zr Enamel A1, Kuraray Noritake Dental Inc., Tokyo, Japan) was applied to the labial surface and sintered (VITA Vacumat 6000, VITA Zahnfabrik, Bad Säckingen, Germany). After sintering at 940 °C for 1 min under vacuum, the restorations were finished to achieve a 1 mm thickness on the labial surface. The thickness was verified using a caliper. 

For the TL group, a 0.5 mm dentin ceramic (CZR Cerabien Zr Dentin A1, Kuraray Noritake Dental Inc., Tokyo, Japan) was applied to the labial surface and sintered. The labial surface was finished using diamond burs, and a uniform space of 0.3 mm was created for the enamel ceramic. The enamel layer (the same as for the BL group) was applied in a 0.5 mm thickness and sintered at 940 °C for 1 min under vacuum. After the firing procedure, the labial surfaces of the crowns were finished to achieve a thickness of 1 mm.

All crowns were cleaned using a steamer and air-dried. Then, a thin glaze layer (CZR Cerabien Glaze Paste Clear, Kuraray Noritake Dental Inc., Tokyo, Japan) was applied, covering the entire surface of the crowns, and they were fired at 930 °C. Stains or ceramic effects were not used for any of the crown groups. One experienced master dental technician performed all laboratory procedures (Figure 2).

The following zirconia crown groups resulted in: monolithic (ML, n = 5), bi-layer (BL, n = 5), and tri-layer groups (TL, n = 5) all having 1 mm labial and 1.5 mm incisal thickness.

### 2.2. Substrate Fabrication

A polyethylene foil was heated until soft using a Bunsen burner and adapted over the prepared tooth. After cooling, the plastic mold was detached, and composite resin (IPS Natural Die Material, Ivoclar Vivadent, Schaan, Liechtenstein) was densely packed to obtain the duplicate resin dies. The resin was polymerized for 40 s using a light-curing lamp (1200 mW/cm^2^, Halo, Translux Wave, Kulzer, Hanau, Germany). Nine tooth-colored resin substrates were obtained: ND1, ND2, ND3, ND4, ND5, ND6, ND7, ND8, ND9. 

**Figure 3:**
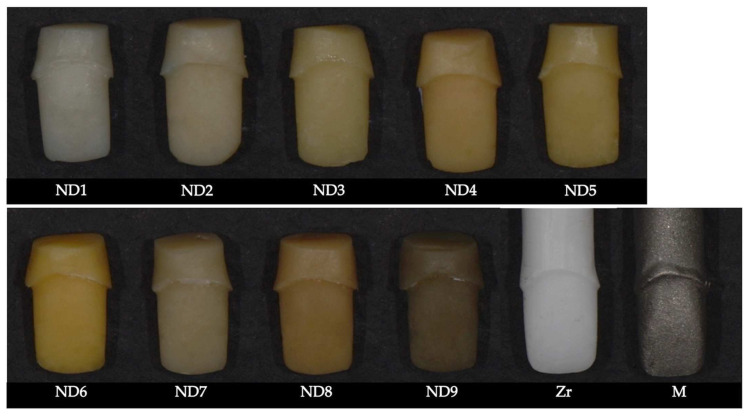
The eleven substrates made of resin composite (ND1–ND9), zirconia, and metal.

To fabricate the zirconia and the metal dies, the prepared tooth was scanned, and the three-dimensional model of the die was digitally processed using CAD software (InLab 15, Sirona Dentsply Gmbh, Bensheim, Germany) for preparing the virtual die for milling and additive manufacturing. For the milling process of the zirconium oxide die, the virtual die was imported in a generic CAM software (SUM3D, CIMsystem, Cinisello Balsamo, Italy), the milling strategy was configured, and the milling process was performed by using a 5-axis milling machine (Coritec 250i, Imes iCore Gmbh, Eiterfeld, Germany) using a translucent zirconia pre-colored disk (Vita YZ T color, LLL2 medium, VITA Zahnfabrik, Bad Säckingen, Germany). To produce the metal die, selective laser melting (SLM) was employed, which required the importation of the virtual die into a specific CAM software (CAMbridge, 3Shape A/S, Copenhagen, Denmark) and the exportation of the three-dimensional printing strategy to the SLM printer (MySint 100, Sisma, Piovene Rocchette, Italy). A cobalt–chromium alloy was used for the metal die fabrication process (Mediloy S-Co, BEGO Medical GmbH, Bremen, Germany) (Figure 3).

### 2.3. Color Measurements

**Figure 4:**
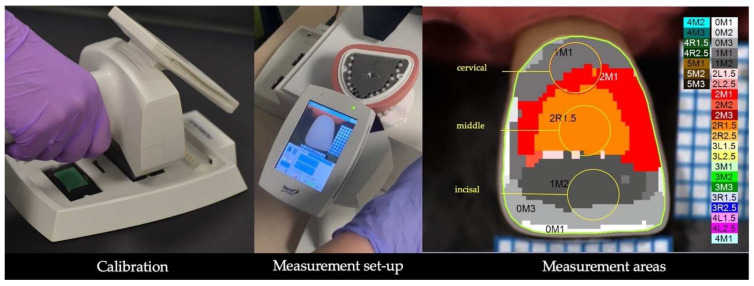
The measurement set-up for the dental spectrophotometer and the data extraction.

Each of the eleven substrates was successively placed into the phantoms’ head dental arch for color measurements. The crowns were seated on the die using a transparent try-in paste (Try-in paste, neutral, Ivoclar Vivadent, Schaan, Liechtenstein), and three color measurements were executed for each crown by a trained operator. The color measurements were performed using a non-contact dental spectrophotometer (Spectroshade Micro, MHT, Niederhasli, Switzerland), and the instrument was calibrated before each measurement using the white and green calibration tiles. The instrument has a CIE 45°/0° illumination/measurement geometry and converts spectral data using the CIE 2° standard observer and a CIE D65 illuminant. The recorded images were transferred to the software’s database, and CIE L*, a*, b*, C*, and h° color coordinates were extracted from three areas (3 mm diameter) of the crown: cervical, middle, incisal. A template was used to ensure the same extraction area for each crown (Figure 4). 

### 2.4. Color Differences and Masking Ability

The masking ability was expressed as the color difference between a crown seated on the ND1 substrate (the control substrate) and the same crown placed over each of the other ten substrates (the test substrates ND2–ND9, Zr, M) [13,33]. The color differences were computed for each measurement area. The CIEDE2000 color difference formula was used for all calculations:

$$\Delta E_{00}={\left[{\left(\frac{\Delta L^{\prime }}{k_{L}S_{L}}\right)}^{2}+{\left(\frac{\Delta C^{\prime }}{k_{C}S_{C}}\right)}^{2}+{\left(\frac{\Delta H^{\prime }}{k_{H}S_{H}}\right)}^{2}+R_{T}\left(\frac{\Delta C^{\prime }}{k_{C}S_{C}}\right)\left(\frac{\Delta H^{\prime }}{k_{H}S_{H}}\right)\right]}^{\frac{1}{2}}$$

where $\Delta L^{\prime }$, $\Delta C^{\prime }$, and $\Delta H^{\prime }$ are the differences in lightness, chroma, and hue, respectively, for the same crown measured over two different substrates. The parametric factors $k_{L}$, $k_{C}$, and $k_{H}$ are correction terms for experimental conditions and were set to 1 in the present study. $S_{L}$, $S_{C}$, and $S_{H}$ refer to the weighting functions that adjust the total color difference considering the location variation of the color difference pair in $L^{\prime }$, $a^{\prime }$, and $b^{\prime }$ coordinates. Finally, the parameter $R_{T}$ is a function (rotation function) that accounts for the interaction between chroma and hue differences in the blue region [20,21].

The masking ability effectiveness was clinically interpreted according to the visual 50:50% perceptibility (PT_00_ = 0.8$\;\Delta E_{00}$ units) and acceptability (AT_00_ = 1.8$\;\Delta E_{00}$ units) color thresholds for dentistry [23], recommended and standardized within ISO/TR 28642:2016 [22]. Furthermore, to evaluate the $\Delta E_{00}$ above the AT_00_, a recent grading system [24] was used. It describes five intervals, where grades 5 and 4 correspond with the PT_00_ and AT_00_, showing an excellent (EM) and acceptable match (AM), respectively. Grades 3, 2, and 1 refer to different mismatch types: moderately unacceptable (MU) when $\Delta E_{00}$ was >1.8 and ≤3.6 $\Delta E_{00}$ units, clearly unacceptable (CU) when $\Delta E_{00}$ was > 3.6 and ≤ 5.4 $\Delta E_{00}$ units, and extremely unacceptable (EU) when $\Delta E_{00}$ was >5.4 $\Delta E_{00}$ units.

The total color difference CIEDE2000 can be divided into the three components: lightness ($\Delta L_{00}$), chroma ($\Delta C_{00}$), and hue ($\Delta H_{00}$) differences, which can be defined as follows [39]:

$$\Delta L_{00}=\frac{\Delta L^{\prime }}{k_{L}S_{L}}\;;\;\Delta C_{00}=\frac{\Delta C^{\prime }}{k_{C}S_{C}}\;;\;\Delta H_{00}=\frac{\Delta H^{\prime }}{k_{H}S_{H}}$$

### 2.5. Statistical Analysis

After performing the Levene’s test of homogeneity of variance (α=0.05) and verifying that equal variances could not be assumed for all CIE color coordinates L*, a*, b*, C*, and h° groups, a Kruskal–Wallis one-way analysis of variance by ranks were applied to evaluate the changes on chromatic coordinates between the different crown groups. The Mann–Whitney U test was applied for the pair-wise comparisons with a Bonferroni correction (level of significance, p<0.001). Contrasts were made between the three crown groups for the same third using the same substrate. The statistical software package used to perform the statistical analysis was SPSS Statistics 20.0.0 (IBM Armonk, New York, NY, USA).

## 3. Results

### 3.1. Color Coordinates

**Figure 5:**
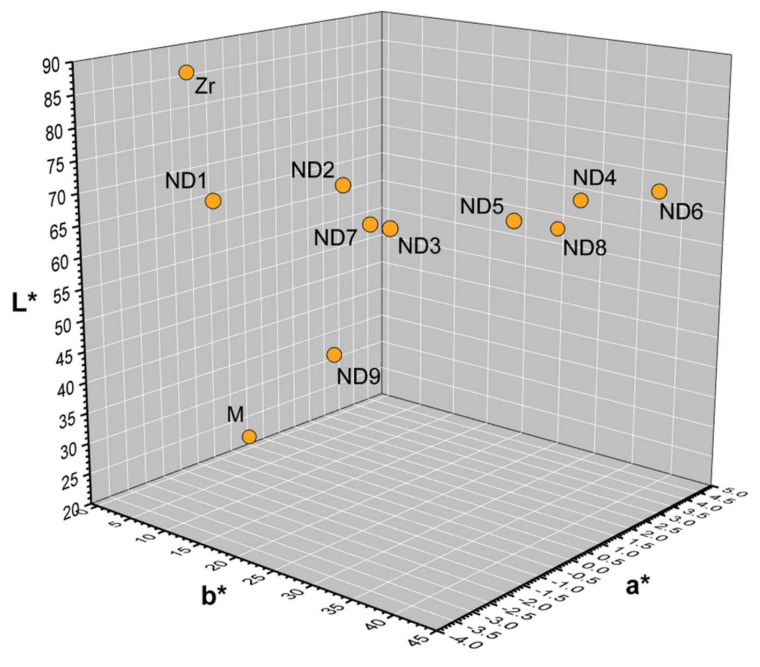
Mean CIELAB values of the eleven substrates.

The distribution of mean CIE L*, a*, and b* values of the substrates are shown in Figure 5. The Zr and M substrates were the brightest and the darkest, respectively. Among the tooth-shaded substrates, ND2 was the brightest and the least chromatic, while ND9 was the darkest, and ND6 was the most chromatic. ND2–ND6 substrates had a comparable lightness to ND1.

CIE L*, a*, b*, C*, and h° color coordinates of the three crown groups measured over different discolored substrates are presented in Table 1, Table 2 and Table 3. 

For ML crowns, the color coordinates ranged between 73.26–84.67 for L*, −2.0–1.28 for a*, 6.74–16.23 for b*, 7.01–16.27 for C*, and 85.33–105.97° for h°. 

For BL crowns, the color coordinates ranged between 71.00–86.24 for L*, −1.71–1.84 for a*, 2.71–14.35 for b*, 2.98–14.42 for C*, and 82.58–116.04° for h°. 

For TL crowns, the color coordinates ranged between 71.51–86.18 for L*, −1.64–1.95 for a*, 4.22–16.05 for b*, 4.41–16.14 for C*, and 83.45–107.06° for h°. 

The L* coordinate did not differ statistically significantly between BL and TL crowns, irrespective of the measurement area or substrate (*p* ≥ 0.001). In the middle area, the L* coordinate of the ML group was statistically different from the BL and TL groups only for Zr and M substrates, while in the incisal area, only for ND7 and M substrates.

The a* coordinate was statistically different between ML crowns and layered crowns (BL and TL) for all measurement areas and substrates, except the Zr abutment. In the cervical area, the b* and C* coordinates differed significantly between the three groups of crowns (*p* < 0.001). However, in the middle and incisal areas, the ML and the TL groups showed similar behavior. The hº coordinate of the ML group differed significantly from layered groups for ND2–ND9 substrates in the cervical area, while in the middle and incisal areas, the BL and TL groups generally showed statistically significant differences among them.

### 3.2. Masking Ability

**Figure 6:**
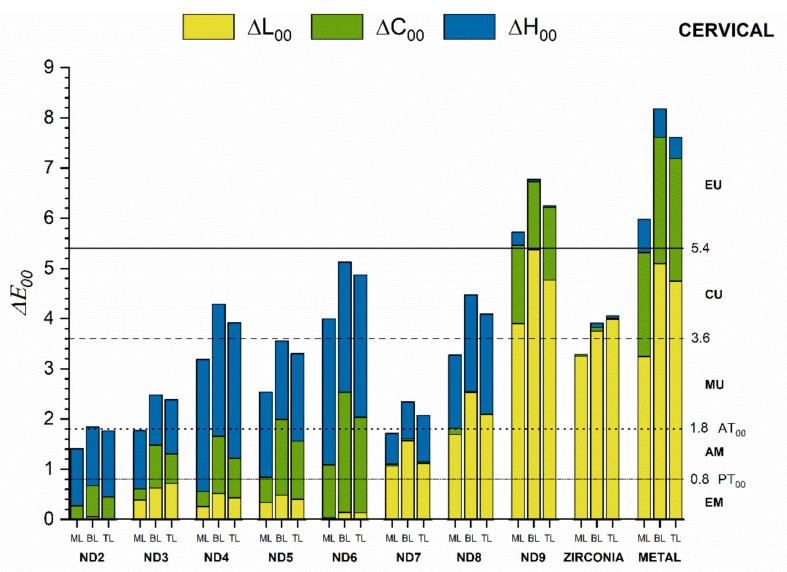
Masking ability of crown groups on different substrates evaluated for the cervical area.

**Figure 7:**
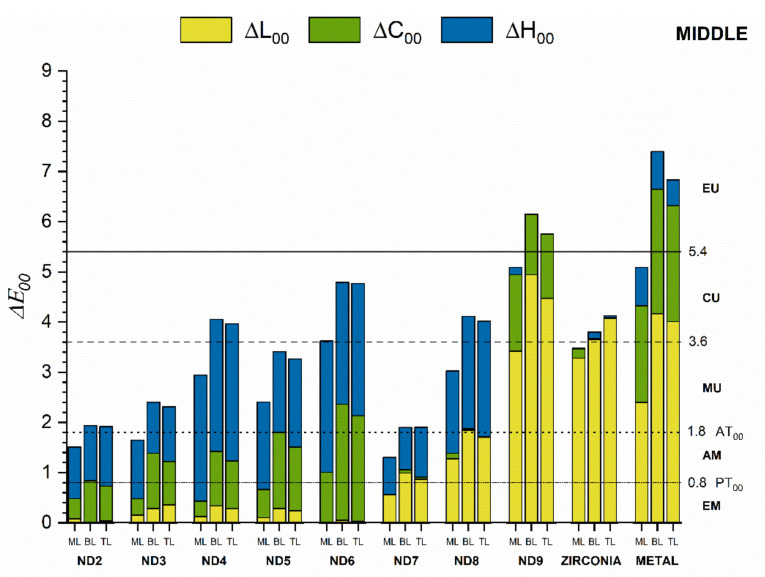
Masking ability of crown groups on different substrates evaluated for the middle area.

**Figure 8:**
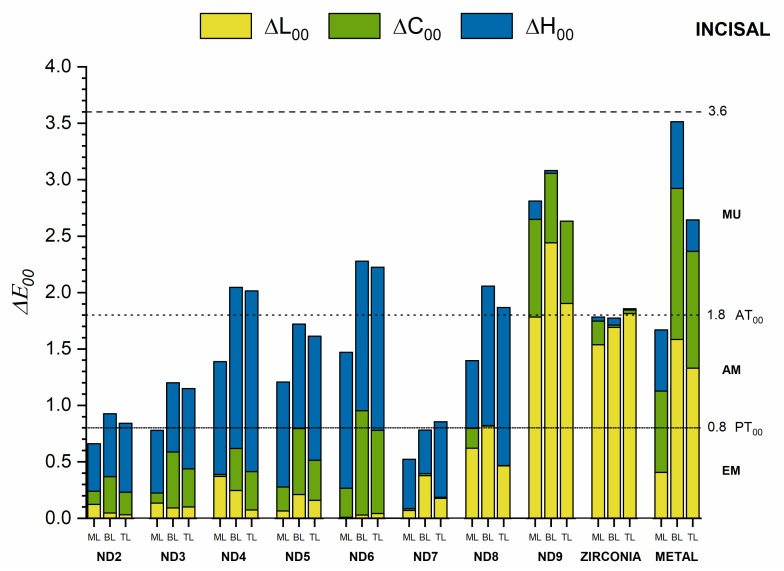
Masking ability of crown groups on different substrates evaluated for the incisal area.

ML had better masking ability than layered crowns regardless of the measurement area or the substrate (Figure 6, Figure 7 and Figure 8). For these crowns, the color differences were below the AT_00_ only for ND2, ND3, and ND7 substrates in the cervical and middle areas and below PT_00_ for the incisal area. A moderately unacceptable color mismatch (MU) was found for ML crowns on ND4, ND5, ND8, and Zr substrates and layered crowns on ND3, ND5, and ND7 substrates for both cervical and middle areas. Nevertheless, for the incisal areas, some of these color differences were acceptable (<AT_00_) (Figure 8).

The lowest masking ability was found for ND9 and M substrates in all areas. In this case, the color differences were extremely unacceptable (EU) in the cervical and middle areas (Figure 6 and Figure 7). For substrates ND2-ND6, the differences in hue (ΔH_00_) and chroma (ΔC_00_) from the ND1 substrate contributed the most to the total color difference. For ND7 and ND8, the differences in lightness (ΔL_00_) and hue (ΔH_00_) had the most significant influence, while for ND9, Zr, and M substrates, the lightness (ΔL_00_) and chroma (ΔC_00_) differences contributed to the most to the color difference. This behavior was found in all three areas of the crowns.

Color differences for the cervical and middle areas were similar and considerably higher than for the incisal area for all crown groups and substrates.

## 4. Discussion

Treatment of localized tooth discoloration is challenging and requires knowledge about the etiology of the lesion and a good understanding of material properties used for treating the tooth defect. The success of the treatment relies on how well the material can hide the discolored substrate and, at the same time, match the color of surrounding dental structures or neighboring teeth [40]. Most esthetic materials (resin composite, dental ceramics) are translucent, and their masking ability (hiding power) of a discolored substrate depends on the severity of the discoloration, the thickness of the restoration, and the level of translucency of the restorative material [12,13,15,16]. 

The present study evaluated the masking ability of monolithic and layered zirconia crowns on eleven different discolored substrates. The design of the study aimed to reproduce the clinical conditions and challenges faced during shade matching of ceramic restorations placed over abutments and tooth preparations of different colors. Comparing the masking ability of crowns fabricated with different technologies (monolithic, layered with enamel, layered with enamel, and dentine ceramics) gave an insight into the colorimetric behavior of these restorations.

In addition, nine tooth-shaded resin dies (ND1–ND9) and two abutment materials (zirconia and metal) were used to simulate various discolorations of prepared teeth. ND1 and ND2 represent the shade of natural dentin; ND3, ND4, and ND5 were more chromatic but comparable in lightness to ND1 and ND2, simulating mild or moderate discolorations. ND6 substrate was the most chromatic, with the highest b* values (the yellowest). ND7 was almost similar to ND2 but less bright, while ND8 and ND9 substrates were the darkest, simulating severe dyschromia. 

Moreover, due to the irregular form of the labial dental surface, the capacity to hide the dyschromic substrate was studied separately along the crown, in the cervical, middle, and incisal thirds. 

The CIE L*, a*, b*, C*, and h° color coordinates were statistically significantly different between the three crown groups generally, regardless of the measurement area or the substrate. Therefore, the first null hypothesis was rejected. However, some similarities in the color coordinates were found. In the cervical area, no statistically significant differences in L*, a*, and h° coordinates were found between BL and TL crowns in general. For the middle and incisal areas, the L* coordinate showed statistically similar values among the three groups of crowns for most substrates, while b* and C* coordinates of ML and TL crowns were statistically similar only in the incisal area. 

We found a masking capacity higher than the AT_00_ in the cervical and middle areas, except for the ML groups on ND2, ND3, and ND7 substrates. Therefore, the second null hypothesis was also rejected. However, the incisal area showed smaller color differences as expected due to the partial influence of the substrate and the consequent increase in translucency. In this area, values above the acceptability threshold (MU) were found for the three groups of crowns only for ND9 and for the BL and TL groups in ND4, ND6, N8, and M substrates, obtaining for the rest of the cases an AM or EM masking ability. Nevertheless, the interpretation of these results should be made with caution since the substrate had a smaller influence in this area.

The darkest substrates (ND9 and M) and the most chromatic substrate (ND6) produced the highest color differences, which were completely or extremely unacceptable (CU or EU) for cervical and middle areas and moderately unacceptable (MU) for incisal areas. It is important to note that as the substrate was darker (Figure 5), the total color difference was mainly influenced by the lightness shift, whereas when it was more chromatic, the hue and chroma shifts were higher, increasing in both cases the total color difference in the three areas evaluated. This again highlights the significant influence that the substrate has on the masking capacity [13,14,17,37].

The best masking effect of the crowns was achieved for ND2, ND3, and ND7 substrates in all thirds, since these substrates were the closest to ND1 (Figure 5), requiring a lower masking ability. The masking ability was acceptable (AM) for the ML crowns and moderately unacceptable (MU) for BL and TL crowns for the cervical and middle areas. The color match was excellent (EM) for ML crowns in the incisal area. For ND7 substrate, the lightness had the most significant influence in the color difference, while for ND2 and ND3, the hue; yet this behavior was not observed in the incisal area of the crowns. Although the color differences calculated for these substrates were almost similar, their visual perception by human observers might be judged differently. A previous study [41] showed that observers preferred shades with lower chroma and/or hue difference rather than lower lightness difference when matching shade guide tabs to natural teeth.

ML crowns showed better masking ability than BL and TL crowns. This result could be explained by the higher opacity of the monolithic crown. Layered crowns were stratified with glass ceramics, which had considerably higher translucency than 3Y-TZP zirconia. 

One study evaluated the masking ability of indirect restorative systems on tooth-colored resin substrates [13]. The authors concluded that 1.5 mm thick samples of veneered 3Y-TZP zirconia (ceramic layering over zirconia) had a better masking effect than monolithic lithium disilicate, translucent zirconia, hybrid ceramic, or heat-pressed ceramic over translucent zirconia samples. In our study, 1 mm thick restorations were fabricated, and the same 3Y-TZP zirconia was used for the monolithic and the layered crowns. Differences between the results could have been generated by the difference in thickness and type of samples but also because the study of Basegio et al. used different zirconia for the monolithic samples than for the layered samples.

Another study [17] also concluded that bi-layer samples produced significantly lower color differences than monolithic samples on discolored substrates. However, the authors included in the monolithic group materials with higher translucency than 3Y-TZP zirconia (4Y-TZP translucent zirconia, lithium disilicate, leucite-reinforced glass-ceramic, feldspathic ceramic). 

The color differences obtained in our study were higher than the AT_00_ in the cervical and middle areas of the crowns, with few exceptions (monolithic crowns on typical dentin-like substrates or with mild discolorations). This result suggests that 1 mm thick zirconia crowns have insufficient masking ability of moderately or severely discolored substrates at this thickness and in combination with a transparent try-in paste.

In a study evaluating the effect of the direct layering of substrates with high-value composite resins on the masking ability of CAD-CAM materials, the authors concluded that the layering with 0.25 mm opaque resins reduced the color differences for veneered zirconia [37]. However, the authors used 1.8 mm thick restorations, which might involve excessive tooth preparation.

One study [38] evaluated the effect of external surface treatments and abutment shades on the color of high translucency self-gazed zirconia crowns. The authors concluded that the abutment’s color had a more significant influence on the final color of the crown than the type of surface finishing. The darker the abutment tooth, the higher was the color difference. Our results are in agreement with these findings; however, we also observed that when the crowns were evaluated on a zirconia abutment which is very bright, the color differences were also very high, leading to a moderately or clearly unacceptable match.

Our results showed that the color differences calculated for the incisal area of the crowns were lower than in the other two areas evaluated. This can be explained by the lower influence of the discolored substrate in the incisal area since a 1.5 mm tooth reduction was performed.

As a limitation of the present study, only 1 mm thick restorations were evaluated using an instrumental method. More configurations of the preparations should be analyzed; in addition, the results of our study should be validated by studies including human observers to judge the color differences and to relate these results with visual perception.

## 5. Conclusions

Within the limitations of the present study, it was concluded that:

1. Color coordinates of monolithic and layered crowns differed significantly on all substrates.
2. ML crowns showed better masking ability than BL and TL crowns, regardless of the substrate or the tooth area. However, an acceptable match for ML crowns was only found for ND2, ND3, and ND7 substrates in the three areas.
3. ML and layered 3Y-TZP zirconia crowns have insufficient masking ability on moderately or severely discolored substrates at 1 mm thickness.

## Figures and Tables

**Table 1:** Mean values and standard deviation of CIE L*, a*, b*, C*, and h° color coordinates of zirconia crowns on different substrates evaluated for the cervical area.

Cervical	ML					BL					TL				
	L*	a*	b*	C*	h°	L*	a*	b*	C*	h°	L*	a*	b*	C*	h°
ND1	79.84 ± 0.15 ^a^	−1.38 ± 0.04	12.24 ± 0.13	12.32 ± 0.14	96.43 ± 0.13 ^c^	79.89 ± 0.17 ^a^	−1.03 ± 0.11 ^b^	8.96 ± 0.41	9.02 ± 0.42	96.53 ± 0.50 ^cd^	79.79 ± 0.29 ^a^	−1.02 ± 0.18 ^b^	10.27 ± 0.33	10.33 ± 0.33	95.67 ± 0.98 ^d^
ND2	79.78 ± 0.15 ^a^	−0.47 ± 0.04	13.35 ± 0.12	13.35 ± 0.12	92.02 ± 0.18	79.47 ± 0.17 ^b^	−0.03 ± 0.10 ^c^	10.62 ± 0.27	10.62 ± 0.27	90.15 ± 0.55 ^d^	79.56 ± 0.39 ^ab^	0.06 ± 0.13 ^c^	11.62 ± 0.29	11.62 ± 0.29	89.69 ± 0.63 ^d^
ND3	78.67 ± 0.20	−0.34 ± 0.05	13.38 ± 0.21	13.38 ± 0.21	91.45 ± 0.20	78.13 ± 0.29 ^a^	0.03 ± 0.06 ^b^	11.19 ± 0.27	11.19 ± 0.27	89.84 ± 0.33 ^c^	77.93 ± 0.35 ^a^	0.10 ± 0.09 ^b^	12.14 ± 0.27	12.14 ± 0.27	89.52 ± 0.41 ^c^
ND4	78.54 ± 0.27	0.83 ± 0.07	13.89 ± 0.25	13.91 ± 0.26	86.57 ± 0.25	77.77 ± 0.26 ^a^	1.52 ± 0.05 ^b^	12.16 ± 0.37	12.25 ± 0.37	82.86 ± 0.24	77.93 ± 0.21 ^a^	1.47 ± 0.11 ^b^	12.84 ± 0.21	12.92 ± 0.22	83.45 ± 0.43
ND5	78.53 ± 0.25 ^a^	0.15 ± 0.02	14.20 ± 0.16	14.20 ± 0.16	89.39 ± 0.10	78.02 ± 0.23 ^b^	0.66 ± 0.08	12.48 ± 0.38	12.50 ± 0.37	86.98 ± 0.45 ^c^	78.17 ± 0.46 ^ab^	0.75 ± 0.13	13.33 ± 0.34	13.35 ± 0.35	86.80 ± 0.50 ^c^
ND6	79.39 ± 0.35	1.28 ± 0.06	15.64 ± 0.21	15.70 ± 0.21	85.33 ± 0.16	78.74 ± 0.33 ^a^	1.84 ± 0.07 ^b^	14.18 ± 0.40	14.30 ± 0.40	82.58 ± 0.35 ^c^	78.66 ± 0.35 ^a^	1.95 ± 0.17 ^b^	14.90 ± 0.41	15.03 ± 0.43	82.54 ± 0.44 ^c^
ND7	77.90 ± 0.19 ^a^	−0.57 ± 0.02	12.02 ± 0.16	12.04 ± 0.16	92.69 ± 0.08	77.16 ± 0.34 ^b^	−0.09 ± 0.12 ^c^	9.46 ± 0.31	9.46 ± 0.31	90.53 ± 0.69 ^d^	77.63 ± 0.73 ^ab^	0.00 ± 0.10 ^c^	10.49 ± 0.34	10.49 ± 0.34	90.03 ± 0.55 ^d^
ND8	76.51 ± 0.22	0.32 ± 0.08	11.44 ± 0.13	11.44 ± 0.13	88.39 ± 0.41	75.16 ± 0.46 ^a^	1.12 ± 0.08 ^b^	9.26 ± 0.30	9.33 ± 0.30	83.06 ± 0.61 ^c^	75.67 ± 0.55 ^a^	1.09 ± 0.16 ^b^	10.21 ± 0.33	10.27 ± 0.34	83.90 ± 0.74 ^c^
ND9	73.26 ± 0.28	−1.61 ± 0.03	7.68 ± 0.13	7.85 ± 0.12	101.85 ± 0.34	71.56 ± 0.47 ^a^	−0.87 ± 0.13 ^b^	4.93 ± 0.24	5.01 ± 0.25	100.02 ± 0.98	72.23 ± 0.72 ^a^	−0.86 ± 0.08 ^b^	6.14 ± 0.33	6.20 ± 0.32	97.99 ± 1.03
Zr	84.67 ± 0.09	−1.48 ± 0.11 ^b^	12.66 ± 0.18	12.75 ± 0.19	96.64 ± 0.42 ^d^	85.58 ± 0.38 ^a^	−1.35 ± 0.21 ^bc^	8.18 ± 0.51	8.30 ± 0.53	99.35 ± 0.99	85.77 ± 0.34 ^a^	−1.30 ± 0.09 ^c^	9.98 ± 0.58	10.06 ± 0.57	97.43 ± 0.71 ^d^
M	73.69 ± 0.52	−1.93 ± 0.04	6.74 ± 0.27	7.01 ± 0.26	105.97 ± 0.69 ^c^	71.00 ± 0.43 ^a^	−1.24 ± 0.08 ^b^	2.71 ± 0.25	2.98 ± 0.26	114.67 ± 1.08	71.51 ± 0.62 ^a^	−1.28 ± 0.11 ^b^	4.22 ± 0.45	4.41 ± 0.43	107.06 ± 2.17 ^c^

Same superscript letter in the same row indicates no statistical significance (*p* ≥ 0.001)—comparison between crown groups for the same measurement area.

**Table 2:** Mean values and standard deviation of CIE L*, a*, b*, C*, and h° color coordinates of zirconia crowns on different substrates evaluated for the middle area.

Middle	ML					BL					TL				
	L*	a*	b*	C*	h°	L*	a*	b*	C*	h°	L*	a*	b*	C*	h°
ND1	79.65 ± 0.24 ^a^	−1.34 ± 0.03	13.01 ± 0.10	13.08 ± 0.10	95.86 ± 0.15 ^c^	80.67 ± 0.27	−1.22 ± 0.05 ^b^	9.20 ± 0.35	9.28 ± 0.35	97.58 ± 0.17	80.06 ± 0.49 ^a^	−1.14 ± 0.12 ^b^	10.99 ± 0.44	11.05 ± 0.43	95.93 ± 0.80 ^c^
ND2	80.12 ± 0.15 ^a^	−0.44 ± 0.03	14.41 ± 0.08	14.41 ± 0.08	91.74 ± 0.12 ^c^	80.89 ± 0.26 ^b^	-0.28 ± 0.05	11.21 ± 0.44	11.22 ± 0.44	91.45 ± 0.25 ^c^	80.35 ± 0.55 ^ab^	−0.08 ± 0.16	12.89 ± 0.60	12.90 ± 0.60	90.39 ± 0.68
ND3	78.94 ± 0.20 ^a^	−0.32 ± 0.03	14.33 ± 0.07 ^b^	14.33 ± 0.07 ^c^	91.27 ± 0.11 ^d^	79.49 ± 0.39 ^a^	−0.23 ± 0.02	11.77 ± 0.50	11.77 ± 0.50	91.13 ± 0.15 ^d^	78.82 ± 0.58 ^a^	−0.04 ± 0.16	13.31 ± 0.70 ^b^	13.31 ± 0.70 ^c^	90.19 ± 0.64
ND4	78.79 ± 0.35 ^a^	0.77 ± 0.12	14.65 ± 0.10 ^b^	14.67 ± 0.11 ^c^	86.99 ± 0.45	78.98 ± 0.36 ^a^	1.19 ± 0.09	12.38 ± 0.64	12.44 ± 0.65	84.51 ± 0.18 ^d^	78.57 ± 0.41 ^a^	1.40 ± 0.17	14.02 ± 0.59 ^b^	14.09 ± 0.60 ^c^	84.30 ± 0.45 ^d^
ND5	78.96 ± 0.17 ^a^	0.20 ± 0.03	15.04 ± 0.08 ^b^	15.04 ± 0.08 ^c^	89.22 ± 0.12	79.26 ± 0.23 ^a^	0.38 ± 0.05	12.77 ± 0.53	12.77 ± 0.53	88.28 ± 0.20	78.80 ± 0.51 ^a^	0.63 ± 0.18	14.29 ± 0.74 ^b^	14.30 ± 0.75 ^c^	87.50 ± 0.55
ND6	79.62 ± 0.48 ^a^	1.09 ± 0.06	16.23 ± 0.14 ^b^	16.27 ± 0.14 ^c^	86.17 ± 0.20	80.06 ± 0.43 ^a^	1.33 ± 0.11	14.35 ± 0.63	14.42 ± 0.63	84.70 ± 0.23	79.51 ± 0.48 ^a^	1.69 ± 0.18	16.05 ± 0.67 ^b^	16.14 ± 0.68 ^c^	84.00 ± 0.37
ND7	78.43 ± 0.22 ^a^	−0.56 ± 0.03	12.97 ± 0.07	12.98 ± 0.07	92.48 ± 0.14 ^b^	78.70 ± 0.37 ^a^	−0.34 ± 0.07	9.85 ± 0.33	9.86 ± 0.33	91.97 ± 0.43 ^b^	78.23 ± 0.67 ^a^	−0.12 ± 0.14	11.50 ± 0.57	11.51 ± 0.57	90.65 ± 0.70
ND8	76.87 ± 0.32 ^a^	0.41 ± 0.12	12.23 ± 0.10 ^c^	12.24 ± 0.10 ^d^	88.07 ± 0.58	76.75 ± 0.58 ^a^	1.00 ± 0.10 ^b^	9.74 ± 0.40	9.79 ± 0.41	84.13 ± 0.37 ^e^	76.36 ± 0.67 ^a^	1.15 ± 0.18 ^b^	11.27 ± 0.6 1^c^	11.33 ± 0.62 ^d^	84.21 ± 0.59 ^e^
ND9	73.83 ± 0.33 ^a^	−1.44 ± 0.02	8.73 ± 0.09	8.85 ± 0.09	99.40 ± 0.15 ^c^	72.96 ± 0.98 ^a^	−0.88 ± 0.09 ^b^	5.60 ± 0.17	5.67 ± 0.18	98.95 ± 0.84 ^c^	73.00 ± 1.05 ^a^	−0.84 ± 0.07 ^b^	7.19 ± 0.44	7.24 ± 0.43	96.71 ± 0.86
Zr	84.64 ± 0.29	−1.59 ± 0.11 ^bc^	14.27 ± 0.10	14.36 ± 0.10	96.35 ± 0.42 ^d^	86.24 ± 0.27 ^a^	−1.70 ± 0.09 ^b^	8.81 ± 0.47	8.97 ± 0.48	100.92 ± 0.48	86.18 ± 0.40 ^a^	−1.48 ± 0.14 ^c^	11.28 ± 0.48	11.37 ± 0.46	97.50 ± 0.97 ^d^
M	74.76 ± 0.54	−2.03 ± 0.05	7.95 ± 0.28	8.21 ± 0.27	104.36 ± 0.72 ^c^	72.92 ± 0.88 ^a^	−1.50 ± 0.09 ^b^	3.09 ± 0.34	3.44 ± 0.34	116.04 ± 1.29	72.81 ± 1.33 ^a^	−1.52 ± 0.11 ^b^	5.21 ± 0.21	5.43 ± 0.20	106.26 ± 1.41 ^c^

Same superscript letter in the same row indicates no statistical significance (*p* ≥ 0.001)—comparison between crown groups for the same measurement area.

**Table 3:** Mean values and standard deviation of CIE L*, a*, b*, C*, and h° color coordinates of zirconia crowns on different substrates evaluated for the incisal area.

Incisal	ML					BL					TL				
	L*	a*	b*	C*	h°	L*	a*	b*	C*	h°	L*	a*	b*	C*	h°
ND1	77.84 ± 0.30 ^ab^	−1.56 ± 0.04	11.40 ± 0.14 ^d^	11.51 ± 0.14 ^e^	97.79 ± 0.26	78.08 ± 0.39 ^a^	−1.37 ± 0.05 ^c^	8.18 ± 0.32	8.29 ± 0.32	99.52 ± 0.21	77.57 ± 0.27 ^b^	−1.38 ± 0.03 ^c^	11.08 ± 0.69 ^d^	11.16 ± 0.68 ^e^	97.10 ± 0.41
ND2	78.12 ± 0.08 ^a^	−1.21 ± 0.04	11.91 ± 0.16 ^c^	11.97 ± 0.16 ^d^	95.80 ± 0.28 ^e^	78.28 ± 0.20 ^a^	−0.95 ± 0.03 ^b^	9.07 ± 0.34	9.12 ± 0.34	96.00 ± 0.17	77.67 ± 0.36	−0.89 ± 0.04 ^b^	11.79 ± 0.68 ^c^	11.83 ± 0.68 ^d^	94.35 ± 0.39 ^e^
ND3	77.44 ± 0.25 ^a^	−1.10 ± 0.05	11.90 ± 0.13 ^b^	11.95 ± 0.13 ^c^	95.31 ± 0.27 ^d^	77.70 ± 0.29 ^a^	−0.89 ± 0.02	9.41 ± 0.38	9.45 ± 0.38	95.39 ± 0.27 ^d^	77.30 ± 0.26 ^a^	−0.78 ± 0.02	12.15 ± 0.77 ^b^	12.18 ± 0.77 ^c^	93.68 ± 0.29
ND4	76.89 ± 0.18 ^a^	−0.69 ± 0.08	11.73 ± 0.16	11.75 ± 0.16	93.35 ± 0.45	77.14 ± 0.24 ^a^	−0.24 ± 0.04	9.64 ± 0.38	9.64 ± 0.38	91.41 ± 0.24	77.08 ± 0.16 ^a^	−0.09 ± 0.06	12.52 ± 0.79	12.52 ± 0.79	90.41 ± 0.28
ND5	77.61 ± 0.15 ^a^	−0.83 ± 0.01	12.33 ± 0.10 ^c^	12.36 ± 0.10 ^d^	93.85 ± 0.03 ^e^	77.28 ± 0.22 ^b^	−0.61 ± 0.06	9.78 ± 0.36	9.80 ± 0.36	93.54 ± 0.36 ^e^	77.10 ± 0.69 ^ab^	−0.45 ± 0.05	12.37 ± 0.88 ^c^	12.38 ± 0.88 ^d^	92.12 ± 0.35
ND6	77.80 ± 0.19 ^a^	−0.63 ± 0.05	12.53 ± 0.23	12.54 ± 0.22	92.89 ± 0.30	77.91 ± 0.27 ^a^	−0.28 ± 0.06	10.48 ± 0.57	10.48 ± 0.57	91.57 ± 0.40	77.96 ± 0.32 ^a^	−0.13 ± 0.05	13.24 ± 0.64	13.24 ± 0.64	90.56 ± 0.22
ND7	77.72 ± 0.15	−1.20 ± 0.03	11.48 ± 0.04 ^b^	11.54 ± 0.04 ^c^	95.95 ± 0.16	77.35 ± 0.27 ^a^	−1.01 ± 0.07	8.39 ± 0.26	8.45 ± 0.26	96.83 ± 0.43	77.04 ± 0.30 ^a^	−0.82 ± 0.03	11.32 ± 0.72 ^b^	11.35 ± 0.72 ^c^	94.16 ± 0.30
ND8	76.54 ± 0.20 ^a^	−0.80 ± 0.03	10.85 ± 0.16 ^b^	10.88 ± 0.16 ^c^	94.24 ± 0.16	76.28 ± 0.18 ^a^	−0.23 ± 0.03	8.45 ± 0.35	8.45 ± 0.35	91.54 ± 0.26	76.27 ± 0.26 ^a^	−0.16 ± 0.04	11.27 ± 0.75 ^b^	11.27 ± 0.75 ^c^	90.82 ± 0.25
ND9	74.73 ± 0.14 ^a^	−1.70 ± 0.02	8.99 ± 0.13 ^b^	9.15 ± 0.13 ^c^	100.70 ± 0.26 ^d^	74.28 ± 0.80 ^a^	−1.23 ± 0.05	6.33 ± 0.28	6.45 ± 0.28	101.00 ± 0.38 ^d^	74.47 ± 0.37 ^a^	−1.12 ± 0.04	9.10 ± 0.70 ^b^	9.17 ± 0.69 ^c^	97.06 ± 0.50
Zr	80.21 ± 0.16 ^a^	−1.55 ± 0.11 ^bc^	12.36 ± 0.14 ^d^	12.46 ± 0.14 ^e^	97.15 ± 0.48 ^f^	80.57 ± 0.32 ^a^	−1.61 ± 0.03 ^c^	8.14 ± 0.23	8.30 ± 0.23	101.20 ± 0.22	80.19 ± 0.36 ^a^	−1.46 ± 0.06 ^b^	11.38 ± 0.74 ^d^	11.47 ± 0.73 ^e^	97.35 ± 0.66 ^f^
M	76.70 ± 0.25	−1.99 ± 0.04	9.56 ± 0.11	9.77 ± 0.11	101.77 ± 0.30	74.80 ± 0.69 ^a^	−1.71 ± 0.10 ^b^	4.96 ± 0.49	5.25 ± 0.49	109.06 ± 0.99	75.03 ± 1.00 ^a^	−1.64 ± 0.06 ^b^	8.61 ± 0.31	8.77 ± 0.30	100.78 ± 0.76

Same superscript letter in the same row indicates no statistical significance (*p* ≥ 0.001)—comparison between crown groups for the same measurement area.

## Data Availability

The data presented in this study are available on request from the corresponding author.
